# Sacral Reflex Characteristics of Patients with Multiple System Atrophy

**DOI:** 10.1155/2020/6167989

**Published:** 2020-06-27

**Authors:** Zhifang Pan, Xueming Zhang, Xun Wang, Binbin Deng, Wanli Zhang, Huanjie Huang

**Affiliations:** ^1^First Affiliated Hospital of Wenzhou Medical University, Wenzhou 325000, China; ^2^First People's Hospital of Ning Yang, Taian 271000, China

## Abstract

**Objectives:**

To observe and analyze the parameters of the sacral reflex and pudendal nerve somatosensory evoked potential (SSEP) in patients with multiple system atrophy (MSA) with respect to factors such as age, disease course, and subtype and provide evidence for the clinical diagnosis of MSA.

**Materials and Methods:**

A total of 51 MSA patients and 30 healthy controls were selected from the First Affiliated Hospital of Wenzhou Medical University from May 2013 to November 2015. Electrophysiological sacral reflex detection and SSEP detection were performed using the Keypoint EMG/EP system. The extraction rate, latency, and amplitude of the sacral reflex and SSEP in the MSA group and control group were compared.

**Results:**

The sacral reflex latency and amplitude in patients with MSA were statistically different from those of the healthy controls. The latency of sacral reflex increases with the prolongation of the disease course, and the amplitude and initiation rate decrease with the prolongation of the disease course. There was no significant difference in sacral reflex latency and amplitude between MSA patients of different ages and subtypes. There was no significant difference in the latency or amplitude of SSEP between the MSA group and healthy control group.

**Conclusions:**

The latency of sacral reflex increases with the prolongation of the disease course, and the amplitude and extraction rate decrease with the prolongation of the disease course. There was no significant difference in the parameters of sacral reflex between young MSA patients and elderly patients. And there was no statistically significant difference between MSA-P subtypes and MSA-C subtypes. This trial is registered with ISRCTNCR2009041.

## 1. Introduction

Multiple system atrophy (MSA) is a sporadic and progressive neurodegenerative disease of unknown cause that has adult onset. It is divided into two types: multiple system atrophy-Parkinsonism (MSA-P) and multiple system atrophy-cerebellar (MSA-C) with cerebellar ataxia as the main clinical symptom. Early abnormal sacral spinal cord function has become an independent predictor of disease progression and poor prognosis. Therefore, standard assessment of abnormal sacral spinal cord function is important for the diagnosis and prognosis of MSA [1–3]. Among the objective examination methods, the internationally recognized neurophysiological method for evaluating MSA genitourinary dysfunction is external anal sphincter electromyography (EAS-EMG). Because EAS-EMG can reflect the function of Onuf's nucleus and indirectly reflect urine and reproductive function, it can be used to assess sacral spinal cord function [4–7]. Different degrees of neurogenic damage can been seen on the EAS-EMG of MSA patients, such as prolongation of the motor unit action potential (MuAP) time limit, increased amplitude, and satellite potential [8, 9]. EAS-EMG was included in the first version of the consensus standard as a secondary diagnostic tool. However, further studies have shown that a negative EAS-EMG result cannot rule out the possibility of MSA, especially in the early stages of the disease [10–12]. Therefore, in the second version of the consensus standard, EAS-EMG was not used as a means of diagnosis. Moreover, EAS-EMG still has some problems. For example, the satellite potential on EAS-EMG in MSA patients is currently considered to be a characteristic change, but satellite potential identification is difficult. Therefore, EAS-EMG operators need to have superb operational skills. Additionally, EAS-EMG is a needle electrode inspection that is invasive. It often takes tens of minutes; therefore, patients do not always consent to examination.

Clinically, squeezing the glans penis can induce the bulbocavernosus reflex (BCR). By stimulating the pudendal nerve, the BCR is transmitted from the pudendal nerve through the sacral cord S2–S4, which eventually causes contraction of the bulbocavernosus and anal sphincter muscles. The BCR reflects the integrity of the reflex arc consisting of the surrounding afferent nerve, sacral cord, and the surrounding motor fibers. It is a reflection that is closely related to the rectum, bladder, and sexual function. Bors and Blinn et al. [13] used this reflex in 1959 when examining patients with neurogenic bladder. Our neuroelectrophysiology laboratory has established normal values for the BCR in healthy Chinese adults and confirmed that BCR can diagnose the location of neurological diseases by many studies [14, 15]. The nerve impulse generated by stimulating the pudendal nerve, after passing through the spinal cord to the cerebral cortex, produces a potential that can be recorded using an electrode located on the scalp. This potential is called the pudendal nerve somatosensory evoked potential (SSEP). In addition to the integrity of the central pathway, the latency and amplitude of the SSEP are also related to the function of the pudendal nerve. Therefore, the BCR combined with the SSEP can diagnose the location of lesions. This study explored the value of the BCR and SSEP in the diagnosis of MSA by analyzing the characteristics of the BCR and SSEP of MSA patients.

## 2. Materials and Methods

### 2.1. Subjects

A total of 51 MSA patients were admitted to the Department of Neurology of the First Affiliated Hospital of Wenzhou Medical University from May 2013 to November 2015, including 27 men and 24 women. They were aged 45–86 years old, with an average age of 54.79 ± 7.59 years. Disease duration ranged from 6 months to 15 years with an average of 3.08 ± 3.21 years. All MSA patients met the MSA clinical diagnostic criteria [16]. All patients conformed to one of the two inclusion criteria: progressive, sporadic, adult-onset (after age of 30) disease characterized by urinary incontinence plus erectile dysfunction in males or orthostatic decreases in blood pressure and poorly levodopa-responsive Parkinsonism (e.g., bradykinesia with rigidity, tremor, or postural instability), or a cerebellar syndrome (e.g., gait ataxia with cerebellar dysarthria, limb ataxia, or cerebellar oculomotor dysfunction). We excluded patients with a history of diabetes, pelvic surgery, spinal cord diseases, lumbar spine lesions, or lumbosacral radiculopathy based on clinical manifestations, medical history, or imaging examinations. Males with prostatic diseases were also excluded. Thirty healthy subjects were collected from the physical examination center of the First Affiliated Hospital of Wenzhou Medical University. All healthy controls underwent thorough clinical and neurologic examinations. Potential participants with a history of Parkinsonism, gait disturbance, disequilibrium, abnormal sacral spinal cord functions, or neurologic diseases (e.g., epilepsy or stroke) were not included. Magnetic resonance imaging (MRI) scans were performed to confirm that none of the controls had space-occupying or cerebrovascular lesions. All participants provided written informed consent and agreed to participate in the study. The Ethical Decision Committee of the Research Administration at The First Affiliated Hospital of Wenzhou Medical University approved the study (CR2009041).

### 2.2. Measurement Methods

#### 2.2.1. General Information

Neurology physicians conducted detailed medical history interviews and neurological examinations of the participating members. The participants were divided into an MSA group and healthy control group according to clinical manifestations and signs combined with international diagnostic criteria. MSA patients were divided into two groups based on duration of disease, a disease duration of ≤2 years group and a disease duration of >2 years group. At the age of 65, the MSA group and control group were divided into an elderly subgroup and young subgroup.

#### 2.2.2. Testing of the Bulbocavernosus Reflex (BCR) and Pudendal Nerve Somatosensory Evoked Potential (SSEP)

The neurophysiological test was performed using a Keypoint EMG/EP system (Dantec, Bristol, UK). The test required a quiet room and the temperature was controlled at approximately 24°C. The subject's skin temperature was maintained above 32°C and the subject cooperated with the inspection and remained relaxed.

*(1) BCR Test*. The subject was placed in the lithotomy position and the ground line was attached to the ankle. When measuring female subjects, the stimulating cathode was placed on the clitoris, and the anode was placed on the labia. For male subjects, the stimulating electrode was a ring-shaped electrode placed on the scapus penis. A concentric needle electrode was used as the recording electrode, and the electrodes were sequentially inserted into the left and right corpus cavernosum muscles. The electrode impedance was checked before the start of stimulation, and the electrode impedance was required to be <5 kΩ. The stimulation was started at 1.9 square waves per second and the stimulation intensity was set to seven times the sensory threshold. Scan time was set to 5 ms per division, analysis time was set to 0.1 s, and the bandwidth was set to 10–2000 Hz. After recording 20 BCR waveforms, the initial latency and peak-to-peak amplitude of the waveform were calculated and their average values were used in analyses.

*(2) SSEP Test*. The female stimulating electrode was a saddle-like surface electrode placed at the pubic symphysis and the male stimulating electrode was a ring-shaped electrode placed on the scapus penis. The recording electrode was placed at Cz-2 and the reference electrode was placed at Fz. The stimulation intensity was three times the sensory threshold and the frequency of the square wave stimulation was five times per second. The analysis time was 100 ms, and the latency and amplitude of the P40 wave were recorded.

### 2.3. Criteria for Abnormal Electrophysiological Parameters

#### 2.3.1. Criteria for Unilateral BCR Anomalies

1. The value of the patient's unilateral BCR latency was greater than the sum of the mean and 2.5 times the standard deviation of the same-sex control group (*X* + 2.5 SD)
2. The unilateral BCR amplitude of the patient was less than the lowest amplitude of the same-sex control group
3. The patient's BCR waveform could not be elicited
4. BCR abnormality rate = (number of participants with abnormal BCR latency or amplitude (i.e., satisfying condition (1) or (2) or (3))/total number of cases in this group) × 100%
5. BCR unextracted rate = number of cases without BCR/total number of cases in the group × 100%

#### 2.3.2. Criteria for SSEP Anomalies

1. The value of the patient's SSEP latency was greater than the sum of the mean and 2.5 times the standard deviation of the same-sex control group (*X* + 2.5 SD)
2. The amplitude of the SSEP in patients was lower than the lowest amplitude of the same-sex control group
3. The patient's SSEP waveform was not recorded

### 2.4. Statistical Analysis

The data were analyzed using the Statistical Package for the Social Sciences 19.0 (IBM, Armonk, NY, USA). Normal distribution and homogeneity of variance were identified using the Kolmogorov-Smirnov test and Shapiro-Levene's test. The data were normally distributed and are expressed as mean ± standard deviation (*X* ± SD). The sex distribution in the MSA group and healthy control group was compared using the chi-square test and the age and electrophysiological parameters were compared using an independent sample *t*-test. The chi-square test was used to compare the abnormality rate and unextracted rate. *P* < 0.05 was considered statistically significant for all tests.

## 3. Results

### 3.1. Comparison of General Information between Groups (Table 1)

Comparison of general information between groups is provided in Table 1.

### 3.2. Comparison of BCR Test Results between MSA Group and Control Group

#### 3.2.1. Comparison of BCR Parameters between Bilateral Limbs of MSA and Healthy Subjects

There were 51 patients in the MSA group, including 27 male patients and 24 female patients. Bilateral BCR in six male patients and five female patients could not be elicited. There were no significant differences in the BCR latency (*t* = 0.123, *P*=0.902) or amplitude (*t* = 0.326, *P*=0.746) between the left and right sides of the MSA group. The control group comprised 30 members, including 14 men and 16 women; all members had BCR waveforms that could be elicited. Moreover, there was no statistically significant difference between the left and right BCR latency (*t* = 0.2, *P*=0.842) and amplitude (*t* = 0.673, *P*=0.504) in the healthy control group.

#### 3.2.2. Comparison of Mean BCR Latencies and Amplitudes between the MSA and Control Groups (Table 2)

The BCR latency of the MSA group was greater than that of the healthy control group, and the difference was statistically significant (*P* < 0.001) (Table 2). The amplitude of the BCR in the MSA group was lower than that of the control group and the difference was statistically significant (*P* < 0.001) (Table 2).

#### 3.2.3. Effect of Age on BCR Test Results

At the age of 65, the MSA group and normal control group were divided into two subgroups: an elderly subgroup and young subgroup. There were 13 and 38 patients in the elderly and young subgroup of the MSA group, and 4 and 26 members in the elderly and young subgroup of the healthy control group, respectively. There was no significant difference in the BCR latency or amplitude between the elderly and young subgroups in the MSA group. Similarly, there was no statistically significant difference in BCR latency or amplitude between the elderly and young subgroups in the control group.

#### 3.2.4. Influence of Disease Course on BCR Test Results

In the MSA group, there were 27 patients with a disease duration of ≤2 years (14 men, 13 women) and 24 patients with a disease duration of >2 years (13 men, 11 women). The average BCR latency of MSA patients with a disease duration of >2 years was greater than that of patients with a disease duration of ≤2 years. The difference in latency between the two subgroups was statistically significant. The average BCR amplitude of MSA patients with a disease duration of >2 years was lower than that of patients with a disease duration of ≤2 years. The difference between the two subgroups was statistically significant (Table 3).

#### 3.2.5. Comparison of BCR Test Results between MSA-C Subtype and MSA-P Subtype

In the MSA group, there were 23 cases of the MSA-C subtype (12 men, 11 women) and 28 cases of the MSA-P subtype (15 men, 13 women). As shown in Table 4, the BCR latency and amplitude of the MSA-C subtypes were not significantly different from those of MSA-P subtypes (*P* > 0.05).

#### 3.2.6. Comparison of BCR Abnormality Rate and Unextracted Rate in Each Subgroup of the MSA Group

There were 51 cases in the MSA group. Among them, 42 patients had an abnormal BCR, giving a total abnormality rate of 82.35% (42/51). The BCR of 11 patients could not be elicited and the total nonextraction rate was 21.57% (11/51). There was no significant difference in the BCR elicitation rate between the elderly and youth subgroup of MSA group. The elicitation rate of BCR in MSA patients with a disease duration of >2 years was higher than that in MSA patients with a disease duration of ≤2 years; these differences between the two subgroups were statistically significant. The BCR elicitation rate in the MSA-C subgroup was also lower than that in the MSA-P subgroup, but the difference was not statistically significant (Table 5).

### 3.3. Comparison of SSEP Test Results between the MSA Group and Healthy Control Group

Regardless of male or female sex, there was no significant difference in the latency or amplitude of SSEP between the MSA group and healthy control group (*P* > 0.05).

### 3.4. Clinical Features of Sacral Spinal Cord Damage and Electrophysiological Test Results

#### 3.4.1. Clinical Symptoms of Abnormal Sacral Spinal Cord Function in the MSA Group

The clinical symptoms of abnormal sacral spinal cord function in the MSA group mainly included nonfluent urination, defecation disorder, and orthostatic hypotension, and the incidence rates were 58.8%, 47.1%, and 45.1%, respectively. In addition, the incidence of sexual dysfunction in male patients was 52.9%. Thus, urinary dysfunction and male sexual dysfunction were the most common clinical symptoms.

#### 3.4.2. Comparison of Rates of BCR Abnormality between the MSA with Abnormal Sacral Spinal Cord Function Group and MSA without Abnormal Sacral Spinal Cord Function Group

According to the abnormality judgment criteria, the rates of abnormal BCR parameters in each group were calculated separately (Table 6). The results showed that the rate of BCR abnormality in the MSA with nonfluent urination group was significantly higher than that in MSA without nonfluent urination group and the difference was statistically significant. The rate of BCR abnormality in the MSA sexual dysfunction group was significantly higher than that in the nonsexual dysfunction group and the difference was statistically significant. The rate of BCR abnormality in MSA patients with defecation disorder was higher than that in patients without defecation disorder and the rate of BCR abnormality in MSA patients with orthostatic hypotension was lower than that in patients without orthostatic hypotension; however, the differences were not statistically significant.

## 4. Discussion

Abnormal sacral spinal cord function is not only considered by neurologists as an important clue to the diagnosis of MSA but also one of the necessary conditions for the establishment of MSA in the MSA diagnostic guidelines [7]. Among the various neurological dysfunctions caused by MSA, the inconvenience and declining quality of life of patients with abnormal sacral spinal cord function are most obvious and widespread [17, 18]. Lesions of the intermediolateral cell column of the spinal cord and vagus dorsal nucleus are the main pathological changes in MSA sacral spinal cord dysfunction, and the lesions involve both sympathetic and parasympathetic systems [19]. Neuronal degeneration in Onuf's nucleus located in spinal segment S2–S4 causes damage to the sacral spinal cord innervating the bladder and rectal sphincter, which in turn leads to clinical signs of defecatory and urinary dysfunction. Previous studies have shown [20–24] that about 76% of patients who were eventually diagnosed with MSA complained of symptoms of abnormal sacral spinal cord function when presenting to a doctor. These symptoms primarily consist of constipation, urinary retention, urinary incontinence, impotence, and hypotension associated with postural changes. Therefore, an objective and reliable assessment of abnormal sacral spinal cord function is critical to the diagnosis and treatment of MSA patients. This study found that both MSA-P and MSA-C patients had impairment of sacral spinal cord function, mainly characterized by urinary dysfunction, sexual dysfunction, and orthostatic hypotension, which is consistent with previous studies. At the same time, our electrophysiological examination results show that BCR abnormalities can occur in early MSA, and these abnormalities may be related to damage of the sacral spinal cord.

The reflex arc of the bulbocavernosus reflex (BCR) involves two components. The first component has an incubation period of about 35 ms, which is clinically useful. The second component is a late response with an incubation period of about 60–70 ms, which has not been used in clinical practice and is easily induced only in patients with lesions above the sacral cord. Our neuroelectrophysiology laboratory has conducted extensive research on the BCR. Niu et al. [25] found that women with abnormal urethral sphincter function had a longer BCR latency and lower BCR amplitude. Wang et al. [26] have taken the lead in establishing the normal value of BCR in healthy adults in China and have confirmed through many studies that the BCR can be of great value in locating and diagnosing neurological diseases. The BCR is a reflex that is closely related to the rectum, bladder, and sexual function. The results of this study showed that, compared with the control group, the BCR latency of the MSA group was prolonged and the amplitude was decreased. The rate of BCR abnormality in MSA patients was 82.35% (42/51), while the BCR in all members of the control group was normal. The SSEP amplitude and latency of the MSA patients were not statistically different from those of the control group, which was consistent with previous domestic studies. This result indicates that the integrity of the BCR reflex arc in MSA patients is impaired. The BCR reflex arc includes the pudendal afferent nerve, sacral cord S2–S4, pudendal efferent nerve, and finally bulbocavernosus muscle contraction. Combined with the SSEP results, it can be seen that the site of damage in the reflex arc in MSA patients is in the sacral cord S2–S4 or pudendal efferent nerves. At present, most neurologists believe that degeneration of the spinal Onuf's nucleus is the main cause of MSA urinary dysfunction and impotence [27]. Onuf's nucleus is a motor nucleus located in the gray matter of the sacral cord S2–S4; we speculate that the lesion causing BCR abnormality in MSA patients is in Onuf's nucleus. Our further studies have found that MSA patients with urinary dysfunction have a higher rate of BCR abnormality than MSA patients without urinary dysfunction, and MSA patients with sexual dysfunction have a higher rate of BCR abnormality than those without sexual dysfunction. It is suggested that there may be a significant correlation between BCR and urinary dysfunction and sexual dysfunction in MSA patients. Wang et al. [10] found that MSA patients with urinary dysfunction had higher rates of abnormal EAS-EMG than those without corresponding symptoms, which was similar to the results of this study. In the study, we found that the rate of BCR abnormality in MSA patients with orthostatic hypotension was lower than that in patients without orthostatic hypotension, but the difference was not statistically significant. Previous studies have shown that the order of abnormal sacral spinal cord function symptoms is sexual dysfunction, urinary dysfunction, and orthostatic hypotension [17, 28–30]. However, in this study, patients with MSA without urinary dysfunction or sexual dysfunction had abnormal BCR parameters (decreased amplitude or prolonged latency). This indicates that degeneration of Onuf's nucleus occurs in the early stages of MSA. The results suggest that BCR can detect clinical changes caused by MSA-related abnormal sacral spinal cord function. At the same time, this study also showed that although BCR examination can reflect the function of the sacral spinal cord to a certain extent, the specificity and sensitivity of the examination differ for different MSA clinical types. This difference may be related to differences in the pathogenesis of different types of abnormal sacral spinal cord function [31]. It is generally believed that urinary dysfunction and sexual dysfunction is caused by a lesion to Onuf's nucleus and damage to lateral nucleus of the sacral cord, and orthostatic hypotension is because of damage to hypothalamus and injury of the cornu laterale medullae spinalis [32–34]. This study also found for the first time that there was no statistical difference in the BCR latency, amplitude, abnormality rate, or unextracted rate in elderly and younger adults with MSA. Moreover, there was no statistical difference in these BCR parameters between the MSA-C subgroup and MSA-P subgroup. It indicated that age and different subtypes had no effect on the BCR latency, amplitude, abnormality rate, or unextracted rate. It is further suggested that BCR is applicable in MSA patients of any age and any subtype. As the disease progressed, the BCR abnormality rate and unextracted rate increased. This suggests that BCR can detect abnormal sacral spinal cord function in the early stages of MSA and abnormal sacral spinal cord function develops progressively with the disease.

In conclusion, BCR is one of the adjunct measures for evaluating the status of sacral spinal cord function in MSA patients. It can be used to evaluate abnormal sacral spinal cord function in MSA patients of different ages and subtypes. Combining clinical manifestations, neuroimaging, and other ancillary examinations, clinicians can greatly improve the early diagnosis of MSA. This detection method is simple, convenient, and economical. However, the sample size of this study was not large enough and no repeated tests were performed. Large sample, multicenter, repeated studies are still needed to confirm the results of this test.

## Figures and Tables

**Table 1:** Comparison of general information between MSA and control groups.

	No. of patients	Age (years)	Sex (male %)
MSA	51	54.79 ± 7.59	27 (53%)
Control	30	57.16 ± 7.25	14 (47%)
Statistics		*t* = 1.17	*χ* ^2^ = 0.015
*P* value		0.286	0.903

There was no significant difference in sex or age between the MSA and control groups (*P* < 0.05).

**Table 2:** Comparison of mean BCR latency and amplitude between the MSA and control groups.

	Males			Females		
	Latency (ms)	Amplitude (mV)	*n*	Latency (ms)	Amplitude (mV)	*n*
MSA	62.96 ± 4.55	0.21 ± 0.03	21	69.49 ± 6.32	0.21 ± 0.03	19
Control	34.02 ± 4.99	1.21 ± 0.03	14	36.39 ± 6.02	1.28 ± 0.04	16
	*t* = 25.326	*t* = 133.620		*t* = 22.039	*t* = 116.267	
	*P*=0.0001	*P*=0.0001		*P*=0.0001	*P*=0.0001	

**Table 3:** Comparison of mean BCR latency and amplitude between the long course of disease (>2 years) and shorter course of disease (≤2 years) groups in the MSA group.

	Males		Females	
	Latency (ms)	Amplitude (mV)	Latency (ms)	Amplitude (mV)
Duration ≤2 years	57.89 ± 1.41	0.23 ± 0.02	62.49 ± 2.68	0.22 ± 0.03
Duration >2 years	65.33 ± 3.41	0.17 ± 0.01	72.99 ± 4.37	0.17 ± 0.02
	*t* = 10.221	*t* = 6.069	*t* = 7.610	*t* = 6.938
	*P*=0.0001	*P*=0.0001	*P*=0.0001	*P*=0.0001

**Table 4:** Comparison of mean BCR latency and amplitude between the MSA-P and MSA-C groups.

	Males		Females	
	Latency (ms)	Amplitude (mV)	Latency (ms)	Amplitude (mV)
MSA-P	63.02 ± 4.60	0.22 ± 0.02	69.63 ± 6.36	0.21 ± 0.03
MSA-C	62.91 ± 4.62	0.21 ± 0.03	69.32 ± 6.48	0.20 ± 0.04
	*t* = 0.077	*t* = 0.072	*t* = 0.145	*t* = 0.047
	*P*=0.939	*P*=0.943	*P*=0.886	*P*=0.963

**Table 5:** Comparison of the elicitation rates of BCR in each MSA subgroup.

		No. of patients	Available for BCR	Rate of BCR elicitation (%)	*P* value
Age	≤65 years	38	30	78.95	>0.99
	>65 years	13	10	76.92	

Duration	≤2 years	27	25	92.59	0.009
	>2 years	24	15	62.50	

Subtype	MSA-P	28	22	78.57	0.979
	MSA-C	23	18	78.26	

**Table 6:** Comparison of rates of BCR abnormalities in MSA patients with abnormal sacral spinal cord function and without abnormal sacral spinal cord function.

Group	Subgroup	Number of cases	Number with abnormal BCR	Abnormal BCR rate (%)	*χ* ^2^ value	*P* value
Urination disorder	1	30	30	100.00	4.416	0.036
	2	21	19	90.48		

Defecation disorder	1	24	23	95.83	0.318	0.573
	2	27	24	88.90		

Male sexual dysfunction	1	27	27	100.00	2,888	0.019
	2	24	19	79.17		

Orthostatic hypotension	1	23	20	86.70	1.017	0.313
	2	28	27	96.90		

Note: group 1: MSA with abnormal sacral spinal cord function, group 2: MSA without abnormal sacral spinal cord function.

## Data Availability

The dataset analyzed during the current study is available from the corresponding author upon reasonable request.

## Abbreviations

MSA: Multiple system atrophy
BCR: Bulbocavernosus reflex
MSA-C: Multiple system atrophy-cerebellar
MSA-P: Multiple system atrophy-Parkinsonism
EAS-EMG: External anal sphincter electromyography
SSEP: Pudendal nerve somatosensory evoked potential.
